# Food finds its way to a woman’s heart: *Campylobacter jejuni*-associated myopericarditis

**DOI:** 10.21542/gcsp.2017.8

**Published:** 2017-03-31

**Authors:** Manivannan Veerasamy, Craig T. Alguire

**Affiliations:** 1 Spectrum Health Medical Group; 2 Spectrum Health Frederik Meijer Heart & Vascular Institute

## Abstract

*Campylobacter jejuni*-associated myopericarditis (CAM) has been reported infrequently in the literature. We describe a case of immunocompetent young woman presenting with chest pain, with history of recent travel and diarrhea. Evaluation led to diagnosis of myopericarditis associated with this infection. The patient improved with conservative management. The pathogenesis of CAM remains unknown. Patients present with chest pain, heart failure, pulmonary edema and arrhythmias. Diagnostic evaluation includes EKG, cardiac enzymes, echocardiogram, cardiac MRI and stool culture. Conservative management recommended and routine use of antimicrobial therapy is controversial. CAM is a rare but severe complication of *C. jejuni* infection. It should be considered as a diagnosis in patients presenting with chest pain with associated gastrointestinal symptoms.

## Background

*Campylobacter jejuni (C. jejuni)* is the most common cause of culture-proven bacterial gastroenteritis in the world, and in the United States, >1% of the population acquires the infection each year^1^. The extra-intestinal complications including cardiac involvement are uncommon, but appear to be increasing in incidence. *C. jejuni*-associated myopericarditis (CAM) is quite rare and has been reported infrequently in the literature.

## Case Story

A 24-year-old, previously healthy, female with no significant past medical history traveled to Spain for vacation 3 weeks prior to presentation. Upon returning from Spain, she had diarrhea for which she was treated conservatively. Stool culture was sent by primary physician and turned positive for *C. jejuni* antibody (ELISA test). She did not get any antimicrobial therapy as her diarrhea improved. She presented to the hospital with acute onset of chest pain and shortness of breath. She was found to be in acute respiratory failure with pulmonary edema with elevated troponins (Troponin-T was 1.670, normal value of <0.03) and elevated N-terminal pro b-type natriuretic peptide (Pro–BNP with a value of 16,215, normal values of 0-800).

**Figure 1.**
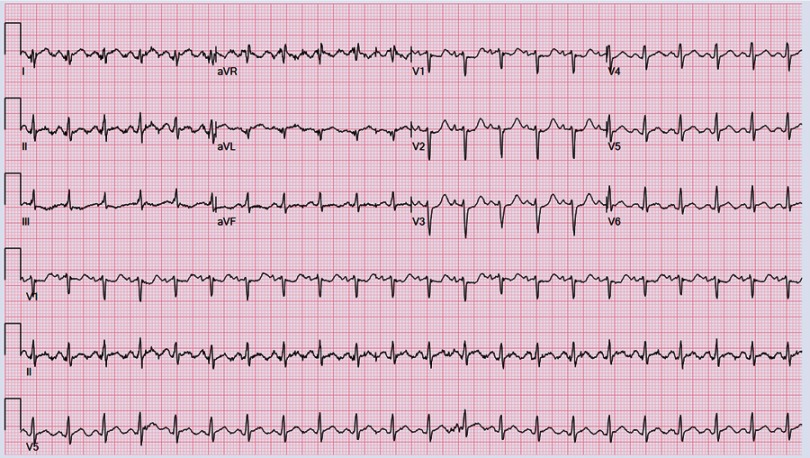
EKG showing sinus tachycardia.

**Figure 2.**
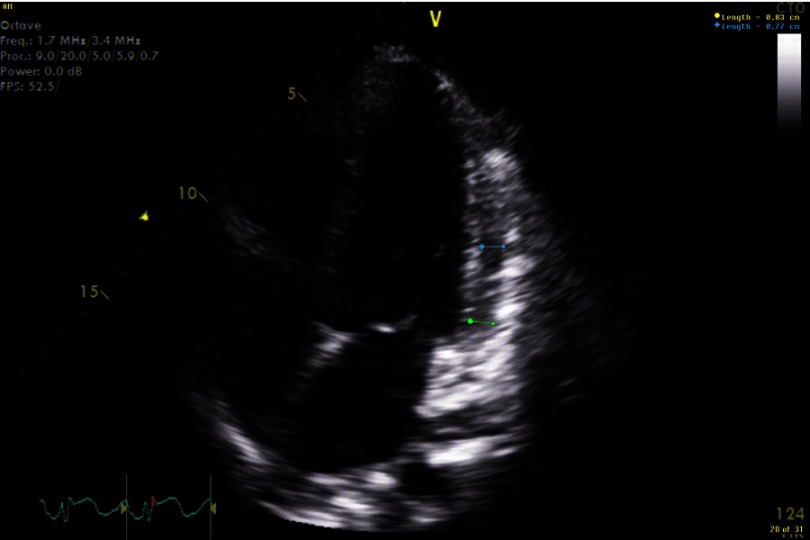
4 chamber view with increased wall thickness.

**Figure 3.**
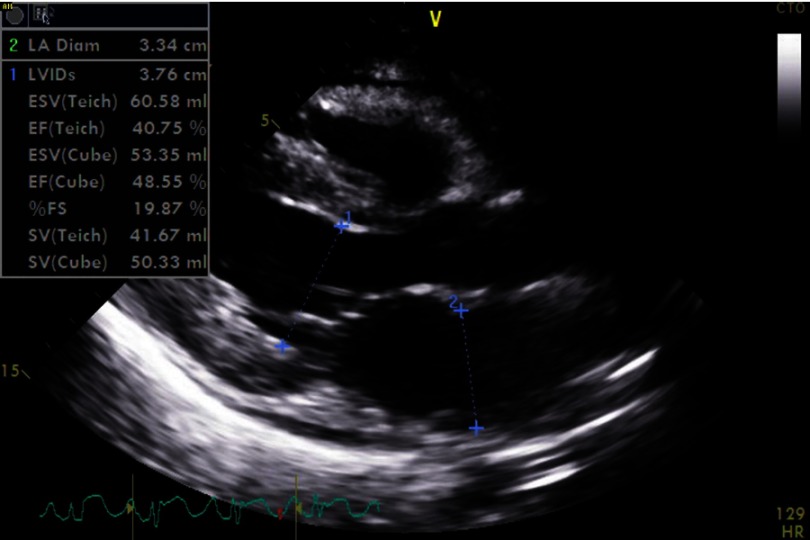
Myocarditis with depressed EF.

**Figure 4.**
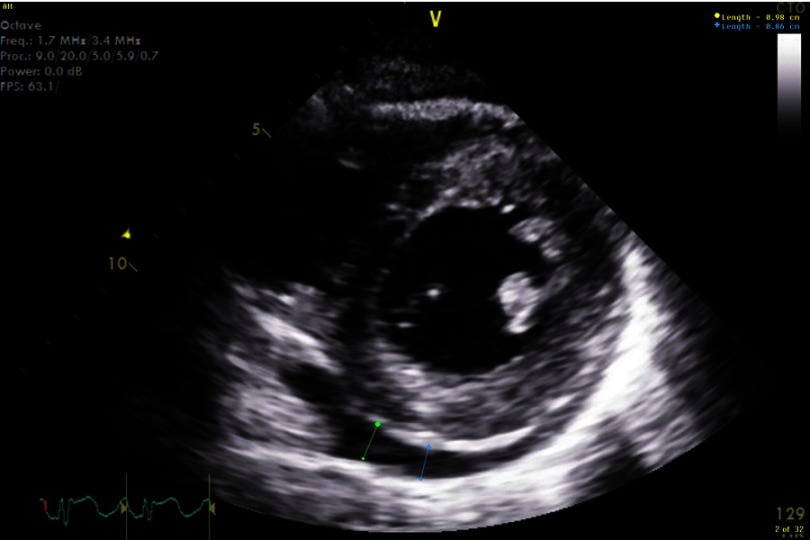
Myocarditis with effusion.

Electrocardiogram (EKG) showed sinus tachycardia, nonspecific ST–T segment changes in the inferolateral leads (Figure 1). Further testing with chest X-ray (CXR) showed bilateral pleural effusions and bilateral infiltrates. Echocardiogram revealed decreased ejection fraction (EF) of 40% with some regional hypokinesis, increased wall thickness and diastolic dysfunction (Figures 2, 3 and 4).

A diagnosis of myopericarditis was made. She was admitted to coronary care unit with continuous cardiac monitoring and was seen by cardiology, pulmonary and infectious disease teams. Extensive workup including bacterial, viral, fungal, autoimmune serologies were performed, all of which came back as negative. It is plausible that this is related to the recent untreated campylobacter infection and probable immune reaction related to this.

Over the next 24 hours of hospital stay, the patient remained hemodynamically stable and troponin levels were trending down. She was treated with diuretics and responded well. Repeat stool culture still came back antibody positive for *C. jejuni* (ELISA test). Though there is no compelling evidence, she was given treatment with anti-microbial therapy. Initially started on levofloxacin, and then switched to Azithromycin (given the high prevalence of quinolone-resistant *C. jejuni* in Spain). She was also treated with beta-blocker, colchicine, and non-steroidal anti-inflammatory medications.

**Figure 5.**
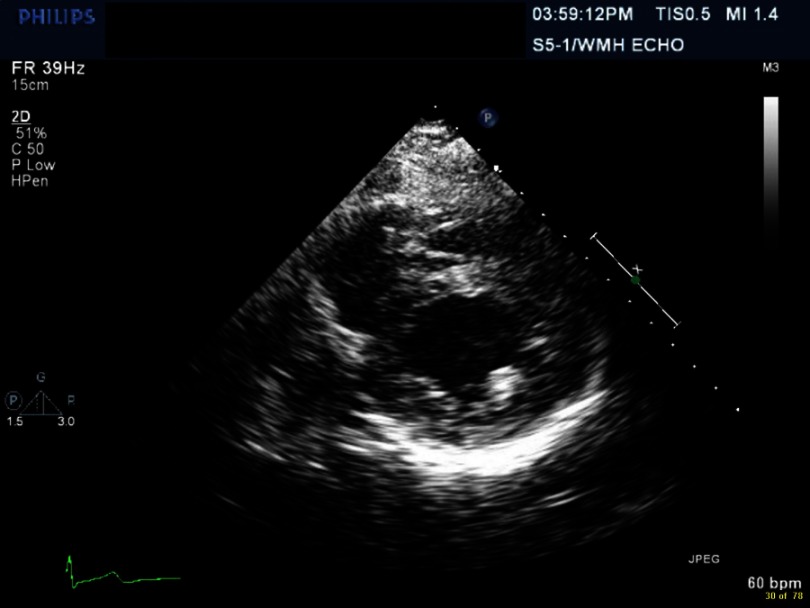
Myocarditis after the treatment.

**Figure 6.**
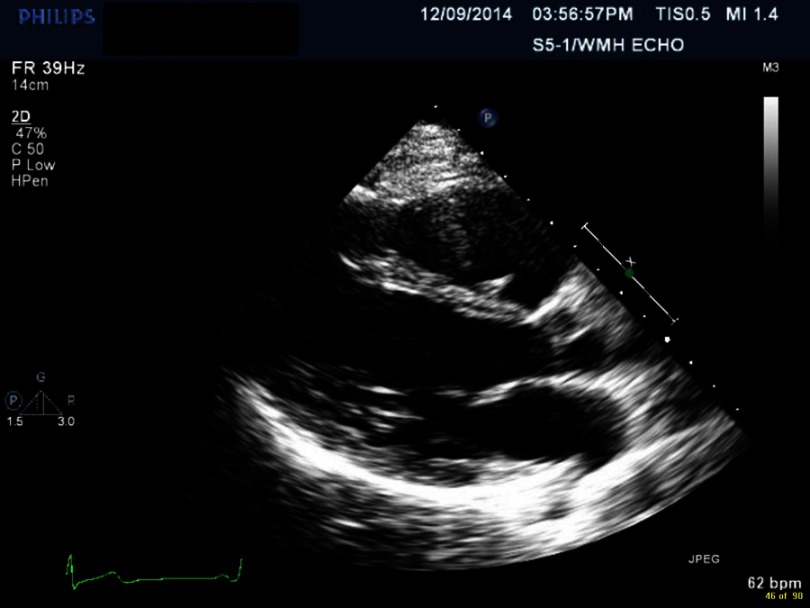
Myocarditis post treatment with improved EF.

**Figure 7.**
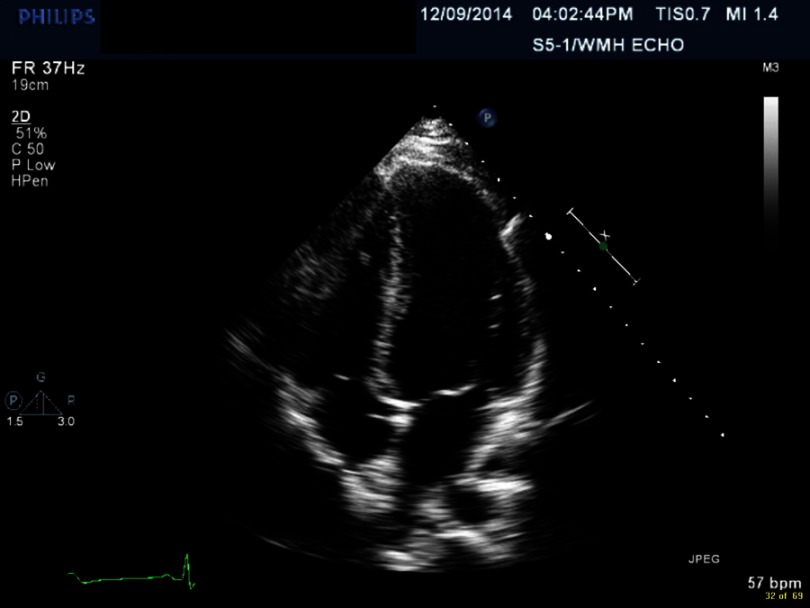
4 chamber view post treatment.

She was discharged home in stable condition after 4 days in the hospital. Follow up echo in 2 weeks showed improved EF of 60% and normal wall motion. Repeat CXR showed complete resolution. Clinically, she improved well and remained completely asymptomatic. Follow up echo after 6 months showed normal EF, wall motion and function (Figures 5, 6 and 7).

## Discussion

Myopericarditis is a challenging clinical diagnosis that involves inflammation of the pericardium, primarily with some involvement of the myocardium^2^. A diagnosis of myopericarditis should be considered when a patient presents with or without chest pain and elevated cardiac enzyme levels, or unexplained congestive heart failure in the absence of coronary disease or coronary spasm.

The etiology of myopericarditis includes idiopathic, infectious, immune-mediated, toxic, and radiation induced. Acute infectious myopericarditis is commonly caused by cardiotropic viruses; enteric bacteria are uncommon pathogens of myopericarditis. *C. jejuni* causes multiple acute and late complications including myocarditis, pericarditis, Cholecystitis, Guillain-Barre Syndrome and reactive arthritis. CAM has been reported quite rarely in the literature.

The exact pathogenesis is not well understood for CAM. There are several theories that have been developed. Possible mechanisms include direct bacterial invasion, toxin and cytotoxic T-cell-mediated immunologic reaction^3^. There are reports of concurrent presentation of gastrointestinal involvement with myocarditis which supports a direct bacterial invasion or toxin-mediated mechanism^4^, though this is not common in cases of *C. jejuni.* Both post infectious autoimmune mechanism or immediate influence on myocytes through either direct damage to cells by bacteria or circulating toxins have been suggested^5^.

The most well-known immunological phenomenon related to *C. jejuni* is the delayed onset of Guillain-Barré syndrome, which is believed to be due to molecular mimicry of surface polysaccharides of *C. jejuni* with gangliosides in nerve tissue. Although *C. jejuni* is known to produce a variety of exotoxins with cytotoxic, hemolytic and hepatotoxic effects, none are known to cause cardiotoxicity^6^. Therefore, the exact mechanism of myopericardial involvement is still unknown. Other reported cardiovascular complications of *C. jejuni* include endocarditis, atrial fibrillation and aortitis with aortic dissection.

Symptoms and signs in patients with myopericarditis are variable depending on whether pericardial or myocardial involvement predominates. The usual clinical presentation involves acute chest pain with electrocardiogram changes, and elevated levels of cardiac enzymes in association with antecedent or coincident enteritis. Myopericarditis may present with a wide spectrum of symptoms, including pleuritic chest pain, recent onset of heart failure from systolic or diastolic dysfunction, atrial or ventricular arrhythmias, cardiogenic shock, or even sudden death^4^.

Diagnosis is usually by a combination of clinical, pathologic, biochemical and imaging features. EKGs show various findings, including mild ST elevation, but lack sensitivity or specificity. Several laboratory studies, including hs-CRP, troponins T and I, CK-MB, brain natriuretic peptide, and leukocyte count are often elevated but nonspecific for the diagnosis.

Echocardiogram is the most commonly used diagnostic tool during the illness, as well as in follow-up. The most common echocardiographic features are nonspecific, including systolic and diastolic dysfunction, segmental wall motion abnormalities (hypokinesia, akinesia, and dyskinesia) that can simulate acute myocardial infarction^7^ and pericardial involvement including effusion.

Cardiac magnetic resonance imaging (CMR), using T2-weighted, early T1-weighted, delayed enhanced images and recently T2 and T1 mapping, has the best diagnostic capability^8^. CMR provides more sensitive and specific information regarding the extent of inflammation, edema of myocardium and pericardial effusion^2^.

Currently, it is the most accurate diagnostic method for both guiding biopsy and following up disease over time^9^. Though endomyocardial biopsy (EMB) remains the gold standard for myocarditis diagnosis, the invasiveness, cost, risks, and lack of availability of EMB in many centers limit its widespread clinical use. Histological Dallas criteria and various other immunohistochemical criteria have been used to confirm the diagnosis. Endomyocardial biopsy is recommended in a few circumstances since acute myopericarditis cannot be reliably diagnosed on clinical grounds or the response to antibiotic and symptomatic therapy is not satisfactory^4^.

Treatment of CAM is usually supportive therapy. Based on the extent of myocardial and pericardial involvement and clinical symptoms, non-steroidal anti-inflammatory agents, colchicine, steroids and cardiac remodeling agents have been used. Other measures, such as activity restriction and follow-up assessments in 6 to 12 months are also recommended.

Given the self-limited nature of most *Campylobacter* infections and the limited efficacy of routine antimicrobial therapy, treatment is warranted only for patients with severe disease meeting several criteria, including extra intestinal infection^1^. At this time, no information is available on the outcome of the use of effective treatment to prevent extra intestinal immunoreactive complications. Most of these patients respond well to treatment and experience complete resolution of symptoms. Prognosis is usually good for most patients, unless there is a significant myocardial involvement, which can cause higher cardiac morbidity and mortality.

We present a case in which a CAM diagnosis was established on the basis of clinical, imaging and laboratory findings; absence of significant other coronary risk factors, age, associated symptoms and travel history, with a positive microbiological culture for *C. jejuni*.

## Conclusion

Myopericarditis is rare but can be a severe complication of infectious diseases, which can potentially lead to cardiomyopathy and congestive heart failure, and should be considered as a diagnosis in patients presenting with chest pain and elevated cardiac enzymes. Although rare, physicians should be vigilant and keep cardiac complications of *C. jejuni* in mind when treating young patients with chest pain as misdiagnosis may result in inappropriate treatment, with potential accompanying complications. Travel history and associated symptoms are valuable tools in early diagnosis.

## Competing interests

None

## LIST OF ABBREVIATIONS

*C. jejuni*: *Campylobacter jejuni*
CAM: *C. jejuni*-associated myopericarditis
EKG: Electrocardiogram
CXR: Chest X ray
ECHO: Echocardiogram
EF: Ejection fraction
EMB: Endomyocardial biopsy
